# Association of dietary inflammatory index with immune-inflammatory biomarkers in rheumatoid arthritis patients: results from NHANES 1999–2018

**DOI:** 10.3389/fnut.2024.1353964

**Published:** 2024-05-27

**Authors:** Zhiming Lu, Zhiheng Xie, Kaiwei Shen, Xunyuan Wu, Eryou Feng

**Affiliations:** ^1^Fujian Medical University Union Hospital, Fuzhou, China; ^2^Department of Orthopaedic, Anxi County Hospital, Quanzhou, China; ^3^Faculty of Medicine, Institute of Biochemistry and Molecular Biology, Albert-Ludwigs-Universität Freiburg, Freiburg, Germany

**Keywords:** rheumatoid arthritis, dietary inflammatory index, systemic immune inflammation index, neutrophil-lymphocyte ratio, platelet-lymphocyte ratio, lymphocyte-monocyte ratio, NHANES

## Abstract

**Background:**

Synovial inflammation is the main reason for joint damage in patients with rheumatoid arthritis (RA). Diet is recognized as one of the therapeutic strategies to control the inflammatory activity in RA. However, few studies have investigated the association between diet and immune-inflammatory biomarkers in RA patients. Our study aims to examine the correlation between dietary inflammatory potential and systemic immune-inflammation Index (SII), neutrophil-lymphocyte ratio (NLR), platelet-lymphocyte ratio (PLR), and lymphocyte-monocyte ratio (LMR) in the RA population.

**Materials and methods:**

The National Health and Nutrition Examination Survey (NHANES) was the data source utilized in this study, spanning from 1999 to 2018. The study encompassed 2,500 RA participants in total. The dietary inflammatory potential was calculated by the dietary inflammation index (DII) score based on dietary recall interviews. The generalized multiple linear regression analyses were used to evaluate the relationship between DII and immune-inflammatory markers. Furthermore, subgroup analyses and restricted cubic spline models were performed.

**Results:**

After full adjustments, there were significant positive correlations between DII levels and SII/NLR in RA patients (SII, β: 14.82, 95% CI: 5.14–24.50, *p* = 0.003; NLR, β: 0.04, 95% CI: 0.01–0.08, *p* = 0.005). It was noteworthy that inconsistent results were observed in the association between DII and SII as well as NLR in subgroups of red blood cell levels (Interaction *p*-value <0.001).

**Conclusion:**

Pro-inflammatory dietary status in the RA population is significantly positively correlated with SII and NLR, influenced by variations in red blood cell levels.

## 1 Introduction

Rheumatoid arthritis (RA) is a systemic inflammatory autoimmune disease, characterized by invasive inflammation that impacts synovial joints with progressive cartilage and bone destruction. Although its etiology remains unknown, the pathogenesis (1) is clear, with continuous immune cell activation induced by immune-mediated mechanisms of RA-specific autoantigens. The formation of a persistent state of inflammation in turn leads to an expansion of the synovial membrane which invades the periarticular cartilage and bone, resulting in joint deformity and disability (2). Therefore, one of the most important measurements of treatment for RA is how relieved the inflammatory activity is. Laboratory inflammatory markers erythrocyte sedimentation rate (ESR) and C-reactive protein (CRP) have been reported to have limited sensitivity and specificity (3), while ultrasound and magnetic resonance imaging are technically dependent and expensive (4). In contrast, some simple biomarkers easily accessible and inexpensive recently have been confirmed to be highly sensitive indicators of systemic inflammatory response. Hu et al. developed the Systemic Immune Inflammation Index (SII) in 2014 (5), which has been used as a prognostic factor in cancer and inflammatory diseases based on three inflammatory biomarkers, including platelets, neutrophils, and lymphocytes. Studies have found a strong positive correlation between SII and RA. Neutrophils and platelets increase relative to lymphocytes under activation of the immune system, resulting in elevated SII, which has been used to predict the risk of rheumatoid arthritis in US adults (6). Likewise, the neutrophil-lymphocyte ratio (NLR), platelet-lymphocyte ratio (PLR), and lymphocyte-monocyte ratio (LMR) are novel reflections of equilibrium between immune-inflammatory biomarkers. NLR and PLR have been reported as predicted treatment feedbacks in RA patients, as a result of the positive correlation with the disease activity and inflammatory parameters (7, 8). Additionally, elevated LMR found in patients with RA than in healthy control subjects means a good value for assessing disease (9).

The limitation of inflammation can help slow down disease progression and sustain joint mobility. However, long-term treatment of anti-inflammatory and anti-rheumatic drugs is not only hampered by severe multisystemic metabolic side effects (10) but is associated with a high financial cost (11). The anti-inflammatory diet is a protective factor (12), which has a positive effect on the onset and development of RA, while the pro-inflammatory diet can contribute to the underlying state of inflammation. Dietary interventions based on vegan diets and polyunsaturated fatty acids could help in reducing RA disease activity (13). A randomized, controlled trial found that four weeks of a controlled vegan diet decreased the number of core immune cells (14). Excessive consumption of red meat, saturated fatty acid and trans fatty acid may exacerbate the inflammation of RA through obesity and the accumulation of white adipose tissue, stimulating the release of pro-inflammatory mediators (15). However, that is still under debate. No significant association between red meat consumption and the risk for RA was found in a recent meta-analysis (16). Moreover, additional sugar intake and excessive energy consumption were widely accepted as risk factors of RA (17). Although controversies existed on the effects of certain foods, it is certain that an inflammatory diet acts as a potential risk factor for the development of RA and its severity.

The dietary inflammatory index (DII) (18) is developed to comprehensively evaluate the overall extent of dietary inflammatory potential. It reflects the standardization of individual intakes to global referent values and has been confirmed to be highly associated with numerous inflammatory diseases. The higher the DII score, the stronger the pro-inflammatory effect, and vice versa. Previous cross-sectional studies have shown that DII levels are higher in RA patients (19, 20). This means they have a stronger pro-inflammatory effect on diets. Until now, the DII has been only applied to analyzed association with disease activity score 28, the high sensitivity C-reactive protein (CRP) and Tumour Necrosis Factor-alpha (TNF-α) in RA (21) and no overall evaluation of this evidence exists. Therefore, the lack of intuitional and credible inflammatory biomarkers to evaluate dietary inflammatory potential might be the main reason why dietary prevention and treatment of RA have not received enough attention. Neutrophil is the most abundant cell type in the inflamed synovial fluid of RA. Diet affects the pathogenesis and progression of RA through modulating the infiltration and activation of neutrophils (17). It has been proved that a one-week total fast (22) and a four-weeks vegan diet (14) improved inflammatory activity in RA patients by reducing the release of leukotriene B_4_ from neutrophils and affecting the number of monocytes and platelets, respectively. Besides, a recent study indicates that both the type and levels of nutrients can influence the generation, survival and function of lymphocytes and therefore can affect several autoimmune diseases (23). As a consequence, there exists a rising interest in whether there is an association between dietary inflammatory potential and these immune-inflammatory biomarkers related to immune cells in RA patients. This study aimed to examine the relationship between DII score and SII, NLR, PLR, and LMR among patients with RA based on a representative sample from the public database, National Health and Nutrition Examination Survey (NHANES).

## 2 Materials and methods

### 2.1 Study population and design

In this study, we utilized data from the NHANES. It is a nationwide cross-sectional study conducted in two-year cycles, collecting and analyzing health and nutrition data for the entire U.S. population through a complex, multi-stage, probability-based sampling weighted design. To increase the sample size of RA patients, we utilized data from multiple cycles spanning from 1999 to 2018. RA was verified using the McQ questionnaire. A total of 100,905 individuals participated in surveys across 10 cycles, with 2,952 self-reported RA patients.

**Figure 1:**
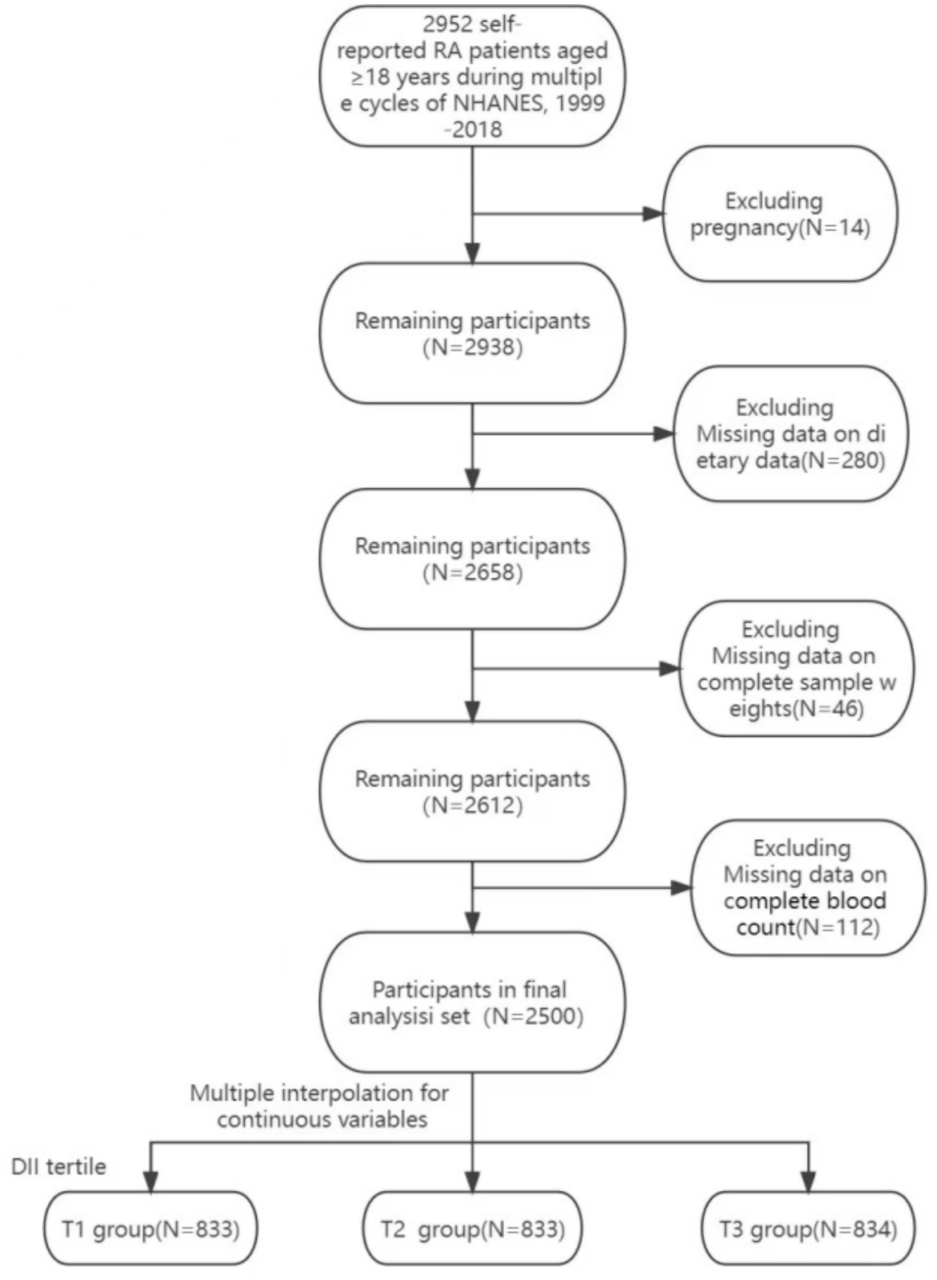
Flowchart of the study design. NHANES, National Health and Nutrition Examination Survey.

For the selection of the study cohort, the following criteria were applied: (I) adults aged 18 and above; (II) participants who self-reported RA, specifying the type of arthritis in response to the question ‘Which type of arthritis was it?’ in the questionnaire; (III) exclusion of pregnant individuals; (IV) inclusion of participants who underwent blood tests, including complete blood cell counts; (V) inclusion of individuals with complete 24-h dietary recall interview data; (VI) incorporation of participants with complete dietary sample weights. A detailed overview of the data selection process is presented in Figure 1. Ultimately, 2,500 individuals were enrolled in this study. This research involved secondary data analysis, preserving the anonymity of individuals and obviating the need for institutional review. This report adheres to the Strengthening the Reporting of Observational Studies in Epidemiology (STROBE) Guidelines (24).

### 2.2 Calculation of the dietary inflammation index

The DII is a composite measure that quantifies the systemic impact of an individual’s dietary intake on inflammation levels within the body. It was developed by Shivappa in 2014 through a comprehensive review of existing literature and was calculated by assigning Z-scores to 45 different dietary components, each with either pro-inflammatory or anti-inflammatory properties. The specific method for calculating the DII aligned with the approach initially proposed by Shivappa and colleagues (18).

Due to the limited availability of specific nutrients in the NHANES dataset, this study computed the overall DII using 28 dietary components, including carbohydrates, proteins, total fats, alcohol, fiber, cholesterol, saturated fatty acids (SFAs), monounsaturated fatty acids (MUFA), polyunsaturated fatty acids (PUFAs), omega-3 and omega-6 fatty acids, vitamins A, B1, B2, B6, B12, C, D, E, folic acid, niacin, magnesium, zinc, iron, selenium, beta-carotene, caffeine, and energy. It’s worth noting that prior research has confirmed that limiting the DII calculation to these 28 nutrients does not affect the DII’s predictive performance (25).

### 2.3 Immune-inflammatory biomarkers (SII, PLR, NLR, LMR)

In our study, SII, PLR, NLR, and LMR were designated as the primary outcome variables. The complete blood count (CBC) parameters in the NHANES dataset from 1999 to 2018 were obtained by analyzing participants’ blood samples using Beckman Coulter equipment (Beckman Coulter, Brea, United States) at mobile examination centers. Platelet, lymphocyte, neutrophil, and monocyte counts were determined using an automated hematology analyzer and were expressed as ×10^3^ cells/μL. The Systemic Immune-Inflammation Index (SII) was computed as follows: SII = (platelet count × neutrophil count)/lymphocyte count (5). Platelet-to-lymphocyte ratio (PLR) and neutrophil-to-lymphocyte ratio (NLR) were calculated by dividing absolute platelet counts and neutrophil counts by absolute lymphocyte counts, respectively. Lymphocyte-to-monocyte ratio (LMR) was determined as absolute lymphocyte counts divided by absolute monocyte counts (26).

### 2.4 Confounding variable

Covariate selection was informed by previously described methods and the inclusion of common confounding variables encountered in clinical practice. Questionnaires identified potential confounders, including age, gender, race, education level, poverty income ratio (PIR), alcohol consumption, and a medical history of conditions like hypertension, diabetes mellitus (DM), congestive heart failure (CHF), stroke, chronic bronchitis, and cancer.

Physical examination data, including body mass index (BMI) and waist circumference, were collected. Laboratory data, such as alanine aminotransferase (ALT), aspartate aminotransferase (AST), red blood cell counts (RBC), creatinine, CRP, and uric acid, were obtained through physiological tests. Gender was categorized as man or woman, while race included groups like Mexican American, other Hispanic, non-Hispanic white, non-Hispanic black, and other races. Education levels were classified as below high school, high school, or above high school. The PIR was calculated by dividing household (or individual) income by the year’s poverty guidelines, leading to three categories: <1.3, 1.3–1.8, and > 1.8. Participants who consumed 4/5 or more drinks daily were considered heavy drinkers. Smoking status was assessed based on serum cotinine levels.

### 2.5 Method of grouping

Under the criteria based on DII tertiles, patients were divided into three groups: T1 (DII < 1.209), T2 (1.209 ≤ DII < 2.834), and T3 (DII ≥ 2.834). For subgroup analysis, patients were further categorized based on the tertiles of continuous variables: age (T1: 20–55, T2: 56–67, T3: 68–85), BMI (T1: 15.20–27.02, T2: 27.03–32.37, T3: 32.38–84.40), and red blood cell counts (T1: 2.78–4.31, T2: 4.32–4.74, T3: 4.75–7.55).

### 2.6 Statistical analysis

All analyses were conducted using R software (version 4.2.1), Stata 16.0, and Em-power Stats software (version 4.1). Sample weights were applied in statistical analyses to ensure representativeness and a significance level of *p* < 0.05 was set to determine statistical significance. Weighted linear regression models were used for continuous variables, and weighted chi-square tests were used for categorical variables to compare the baseline characteristics of the three DII groups. To address missing values for some continuous covariates, multiple imputation was performed.

Generalized weighted multiple linear regression analyses were performed to assess the relationship between the DII and new immune-inflammatory markers in patients with RA while controlling for covariates. Three models were established for multivariate analyses: Model 1 (unadjusted), Model 2 (adjusted for age, gender, race, and BMI), and Model 3 (adjusted for age, gender, race, BMI, waist circumference, education level, poverty-income ratio, drinking status, smoking, hypertension, diabetes, congestive heart failure, stroke, chronic bronchitis, cancer, ALT, AST, RBC, creatinine, and uric acid).

Trend tests were conducted using the median values of the three DII categories. Subgroup analyses were performed based on sample size and *p*-value results, with a particular focus on gender, age, BMI, and the three categories of RBC counts. Subgroup analyses included tests for interactions. Restricted cubic spline (RCS) graphs were used to detect potential nonlinear relationships between major variables, adjusting for covariates, and subsequent subgroup analyses were carried out.

## 3 Results

### 3.1 Baseline characteristics of participant

According to the inclusion criteria, a total of 2,500 participants with RA were included in this study. After applying the appropriate sample weights, the average DII for the entire population was 1.64 ± 1.86. The distribution of participant characteristics across the trisection of the DII was presented in Table 1: T1 (*n* = 833), T2 (*n* = 833), and T3 (*n* = 834). The weighted mean values for the DII tertile were − 0.45 ± 1.21, 2.06 ± 0.46, and 3.58 ± 0.49, respectively. Statistical differences were observed in the proportions of gender, education level, PIR, and the medical history of hypertension, CHF, and stroke among the three groups. This observation suggested that females, individuals with lower educational levels, lower household incomes, and a medical history of hypertension, CHF, and stroke tended to have elevated DII. And there were statistical differences in age (T1: 57.51 ± 14.21 vs. T2: 59.24 ± 14.36 vs. T3: 57.37 ± 14.55 years; *p* = 0.013), BMI (T1: 29.61 ± 7.22 vs. T2: 30.20 ± 6.97 vs. T3: 30.98 ± 7.64 kg/m^2^; *p* = 0.001), Waist (T1: 101.92 ± 16.88 vs. T2: 102.48 ± 16.70 vs. T3: 104.16 ± 16.70 cm; *p* = 0.019), ALT (T1: 27.25 ± 17.30 vs. T2: 25.03 ± 18.63 vs. T3: 23.13 ± 14.45 U/L; *p* < 0.001), AST (T1: 26.77 ± 14.44 vs. T2: 25.73 ± 16.32vs. T3: 24.63 ± 12.92 U/L; *p* = 0.011), RBC (T1: 4.61 ± 0.49 vs. T2: 25.73 ± 16.32vs. T3: 24.63 ± 12.92 million cells/uL; p = 0.011) and CRP (T1: 1.30 ± 2.89 vs. T2: 1.77 ± 3.53 vs. T3: 2.07 ± 4.95 umol/L; p < 0.001) among the three groups. Regarding the primary outcome factors, weighted univariate regression analysis demonstrated a significant association (*p* < 0.05) between higher DII scores and elevated PLR, NLR, LMR, and SII.

**Table 1:** The weighted demographic characteristics of the RA population were stratified by trisection of DII.

Characters	Total (*n* = 2,500)	T1 (*n* = 833)	T2 (*n* = 833)	T3 (*n* = 834)	*p*-value
DII	1.64 ± 1.86	−0.45 ± 1.21	2.06 ± 0.46	3.58 ± 0.49	<0.001*
Age (years)	58.03 ± 14.39	57.51 ± 14.21	59.24 ± 14.36	57.37 ± 14.55	0.013*
Gender (*n*,%)					<0.001*
Man	1,040 (41.51)	433 (53.19)	338 (37.73)	269 (32.16)	
Women	1,460 (58.49)	400 (46.81)	495 (62.27)	565 (67.84)	
Race (*n*,%)					0.093
Mexican American	405 (6.78)	156 (7.6)	140 (6.83)	109 (5.79)	
Other Hispanic	196 (5.29)	59 (4.64)	59 (5.06)	78 (6.26)	
Non-Hispanic White	1,048 (65.71)	359 (67.32)	351 (66.32)	338 (63.24)	
Non-Hispanic Black	725 (16.72)	209 (14.19)	246 (17)	270 (19.3)	
Other Race	126 (5.51)	50 (6.24)	37 (4.79)	39 (5.41)	
BMI (kg/m^2^)	30.24 ± 7.30	29.61 ± 7.22	30.20 ± 6.97	30.98 ± 7.64	0.001*
Waist (cm)	102.81 ± 16.79	101.92 ± 16.88	102.48 ± 16.70	104.16 ± 16.70	0.019*
Education level (*n*,%)					<0.001*
<High school	870 (24.88)	252 (21.26)	301 (25.3)	317 (28.56)	
High school	610 (26.49)	191 (23.93)	200 (26.33)	219 (29.58)	
>High school	938 (44.34)	361 (50.42)	296 (42.23)	281 (39.61)	
Missing	82 (4.29)	29 (4.39)	36 (6.14)	17 (2.25)	
PIR					<0.001*
<1.3	916 (24.55)	263 (24.55)	294 (28.16)	359 (37.51)	
1.3–1.8	341 (12.51)	110 (13.13)	102 (10.78)	129 (13.61)	
>1.8	1,034 (50.68)	378 (53.88)	372 (54.12)	284 (43.46)	
Missing	209 (7)	82 (8.44)	65 (6.94)	62 (5.41)	
Drink status (*n*,%)	484 (20.29)	173 (21.46)	161 (18.87)	150 (20.43)	0.239
Smoke (Serum cotinine, ng/mL)	72.48 ± 137.61	59.47 ± 126.36	63.35 ± 124.38	96.75 ± 158.27	<0.001*
Hypertension (*n*,%)	1,482 (54.16)	473 (51.79)	504 (54.27)	505 (56.73)	0.017*
DM (*n*,%)	612 (19.1)	193 (18.36)	201 (18.37)	218 (20.71)	0.382
CHF (*n*,%)	246 (9.1)	56 (6.39)	87 (9.69)	103 (11.58)	0.001*
Stroke (*n*,%)	237 (8.59)	60 (6.59)	82 (8.94)	95 (10.51)	0.048*
Chronic bronchitis (*n*,%)	309 (15.39)	81 (12.68)	111 (16.63)	117 (17.17)	0.050
Cancer (*n*,%)	398 (17.09)	134 (15.18)	117 (17.11)	147 (19.23)	0.189
ALT(U/L)	25.23 ± 17.01	27.25 ± 17.30	25.03 ± 18.63	23.13 ± 14.45	<0.001*
AST(U/L)	25.76 ± 14.67	26.77 ± 14.44	25.73 ± 16.32	24.63 ± 12.92	0.011*
RBC (million cells/uL)	4.57 ± 0.49	4.61 ± 0.49	4.56 ± 0.49	4.54 ± 0.50	0.011*
Creatinine (umol/L)	72.09 ± 41.11	71.26 ± 33.54	73.00 ± 42.34	72.10 ± 47.17	0.682
Uric acid (umol/L)	332.97 ± 93.89	333.05 ± 88.60	336.50 ± 94.98	329.21 ± 98.37	0.298
Crp (mg/L)	1.70 ± 3.86	1.30 ± 2.89	1.77 ± 3.53	2.07 ± 4.95	<0.001*
PLR	135.00 ± 60.73	132.82 ± 54.18	139.40 ± 65.11	132.91 ± 62.76	0.041*
NLR	2.35 ± 1.46	2.24 ± 1.23	2.43 ± 1.59	2.39 ± 1.56	0.016*
LMR	4.05 ± 1.85	3.97 ± 1.72	3.94 ± 1.79	4.26 ± 2.03	0.001*
SII	606.14 ± 445.95	565.83 ± 386.80	634.69 ± 480.09	622.24 ± 468.01	0.003*

Mean ± SD for continuous variables: *n*,% for categorical variables; *p* value was calculated by weighted linear regression model and weighted chi-square test. * *p* value <0.05. DII, Dietary Inflammatory Index; T1, Tertile 1; T2, Tertile 2; T3, Tertile 3; SD, standard deviation; BMI, body mass index; PIR, Poverty-income ratio; DM, diabetes mellitus; CHF, congestive heart failure ALT, alanine aminotransferase; AST, aspartate aminotransferase; RBC, Red blood cells; CRP, C-reactive protein; PLR, platelet-lymphocyte ratio; NLR, neutrophil-lymphocyte ratio; LMR, lymphocyte-monocyte ratio; SII, systemic immune-inflammation index.

### 3.2 Association between DII and immune-inflammatory biomarkers among RA population

Table 2 displayed the associations between DII and new immunoinflammatory markers (PLR, NLR, LMR, SII) using three weighted generalized linear regression models. In the unadjusted model (Model 1), a significant positive correlation was observed between DII and SII, NLR, and LMR (SII, β (95% CI): 16.25 (6.88, 25.61), *p* < 0.001; NLR, β (95% CI): 0.03 (0.00, 0.07), *p* = 0.028; LMR, β (95% CI): 0.07 (0.03, 0.11), *p* < 0.001). However, in Model 2 and Model 3, where covariates were taken into account, the positive correlation between DII and LMR did not reach statistical significance. Even after adjusting for all potential covariates, SII and NLR retained their statistical significance (SII, β (95% CI): 14.82 (5.15, 24.50), *p* = 0.003; NLR, β (95% CI): 0.04 (0.01, 0.08), *p* = 0.005).

**Table 2:** Associations between DII and immune-inflammatory biomarkers.

Variable	Model 1		Model 2		Model 3	
	β (95%CI)	*p*-value	β (95%CI)	*p*-value	β (95%CI)	*p*-value
DII & SII	16.25 (6.88, 25.61)	<0.001	17.46 (7.88, 27.03)	<0.001	14.82 (5.15, 24.50)	0.003
DII Tertile						
T1	Ref		Ref		Ref	
T2	68.86 (26.66, 111.06)	0.001	68.71 (26.24,111.17)	0.002	57.79 (15.57,100.00)	0.007
T3	56.40 (13.76, 99.05)	0.010	60.59 (17.32,103.87)	0.006	46.77 (3.07, 90.48)	0.036
*p* for trend	0.008		0.005		0.030	
DII & PLR	0.54 (−0.74, 1.82)	0.407	0.60 (−0.71, 1.91)	0.367	0.88 (−0.42, 2.19)	0.185
DII Tertile						
T1	Ref		Ref		Ref	
T2	6.58 (0.83, 12.33)	0.025	6.05 (0.26, 11.84)	0.041	5.47 (−0.22, 11.15)	0.060
T3	0.09 (−5.72, 5.90)	0.976	0.28 (−5.62, 6.18)	0.927	1.21 (−4.67, 7.10)	0.686
*p* for trend	0.902		0.876		0.635	
DII & NLR	0.03 (0.00, 0.07)	0.028	0.05 (0.02, 0.08)	0.002	0.04 (0.01, 0.08)	0.005
DII Tertile						
T1	Ref		Ref		Ref	
T2	0.19 (0.06, 0.33)	0.006	0.21 (0.07, 0.35)	0.003	0.18 (0.05, 0.32)	0.009
T3	0.15 (0.01, 0.29)	0.040	0.21 (0.07, 0.35)	0.004	0.17 (0.03, 0.31)	0.017
*p* for trend	0.034		0.003		0.015	
DII & LMR	0.07 (0.03, 0.11)	<0.001	0.02 (−0.02, 0.06)	0.272	0.01 (−0.03, 0.05)	0.556
DII Tertile						
T1	Ref		Ref		Ref	
T2	−0.03 (−0.20, 0.15)	0.774	−0.11 (−0.28, 0.06)	0.199	−0.09 (−0.25, 0.08)	0.314
T3	0.29 (0.11, 0.46)	0.002	0.09 (−0.08, 0.27)	0.285	0.06 (−0.12, 0.23)	0.532
*p* for trend	0.002		0.313		0.570	

Model 1 No covariates were adjusted; Model 2 Age, gender, race, and BMI were adjusted; Model 3 Age, gender, race, BMI, waist, education level, PIR, drink status, smoke, hypertension, DM, CHF, Stroke, Chronic bronchitis, Cancer, ALT, AST, Red blood cells, Creatinine and Uric acid were adjusted. Ref, Reference; DII, Dietary Inflammatory Index; T1, Tertile 1; T2, Tertile 2; T3, Tertile 3; CI, Confidence Interval; PLR, platelet-lymphocyte ratio; NLR, neutrophil-lymphocyte ratio; LMR, lymphocyte-monocyte ratio; SII, systemic immune-inflammation index.

The models utilized the low DII level (T1) as the reference group. In contrast to the DII T1 group, significant positive associations were observed in all models between SII and NLR and the DII T2 and T3 groups (*p* < 0.05). In the investigation of SII, a stronger positive correlation was evident in T2 compared to the T3 group (T2, β (95% CI): 57.79 (15.57, 100.00), *p* = 0.007; T3, β (95% CI): 46.77 (3.07, 90.48), *p* = 0.036). In examining NLR, the positive correlations in the T2 and T3 groups were relatively close (T2, β (95% CI): 0.18 (0.05, 0.32), *p* = 0.009; T3, β (95% CI): 0.17 (0.03, 0.31), *p* = 0.017). In the DII trend test, SII and NLR showed statistically significant differences in all models. This indicated a significant trend where, with each increase in DII level, the positive correlations decreased.

### 3.3 Regression cubic splines

**Figure 2:**
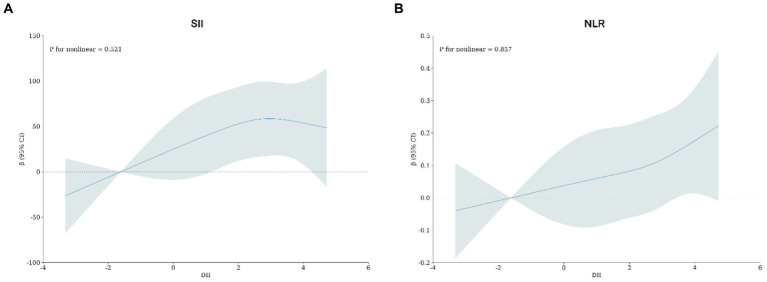
**(A)** Potential nonlinear relationship between DII and SII; **(B)** potential nonlinear relationship between DII and NLR. Adjusted for age, gender, race, BMI, waist, education level, PIR, drink status, smoke, hypertension, DM, CHF, Stroke, Chronic bronchitis, Cancer, ALT, AST, Red blood cells, Creatinine and Uric acid.

Adjusted by Model 3, the restricted cubic spline models revealed that there were no observed nonlinear relationships between DII and SII or NLR in rheumatoid arthritis patients (SII, Non-linear *p* = 0.521; NLR, Non-linear *p* = 0.857; Figure 2).

### 3.4 Subgroup analysis

Subgroup analyses and interaction tests were summarized in Table 3. The association between DII and SII did not show alteration in the stratified analyses based on gender (p for interaction = 0.665), and age tertile (*p* for interaction = 0.284). Although a negative correlation was observed between SII and DII in the BMI tertile, no statistically significant differences were found (T3, β (95% CI): −3.98 (−19.51, 11.55), *p* = 0.616, p for interaction = 0.083). No alteration was observed in the association between DII and NLR when conducting a stratified analysis according to gender (*p* for interaction=0.884), age tertile (*p* for interaction=0.219), and BMI tertile (*p* for interaction=0.240). However, it was noteworthy that inconsistent results were observed in the association between DII and SII as well as NLR in subgroups of red blood cell levels (SII, interaction *p*-value<0.001; NLR, interaction *p*-value<0.001).

**Table 3:** Subgroup analysis.

	β, 95%CI	*p*-value	*p* for interaction
	SII		
Gender			
Man	12.95 (−0.99, 26.88)	0.069	0.665
Women	17.32 (3.63, 31.02)	0.013	
AGE			0.284
Tertile 1	4.23 (−10.60, 19.05)	0.577	
Tertile 2	7.95 (−8.98, 24.89)	0.358	
Tertile 3	34.39 (14.04, 54.74)	0.001	
BMI			0.083
Tertile 1	25.80 (6.57, 45.03)	0.009	
Tertile 2	13.93 (−1.43, 29.30)	0.076	
Tertile 3	−3.98 (−19.51, 11.55)	0.616	
RBC			<0.001
Tertile 1	39.44 (19.51, 59.36)	<0.001	
Tertile 2	13.02 (−1.96, 28.00)	0.089	
Tertile 3	−12.97 (−28.60, 2.66)	0.104	
	NLR		
Gender			0.884
Man	0.05 (0.00, 0.09)	0.047	
Women	0.05 (0.01, 0.09)	0.025	
AGE			0.219
Tertile 1	0.01 (−0.04, 0.06)	0.732	
Tertile 2	0.02(−0.03, 0.07)	0.472	
Tertile 3	0.12 (0.05, 0.18)	0.001	
BMI			0.240
Tertile 1	0.08 (0.02, 0.14)	0.011	
Tertile 2	0.03 (−0.02, 0.08)	0.218	
Tertile 3	0.02 (−0.03, 0.07)	0.516	
RBC			<0.001
Tertile 1	0.12 (0.06, 0.18)	<0.001	
Tertile 2	0.04 (−0.01, 0.08)	0.147	
Tertile 3	−0.04 (−0.09, 0.02)	0.161	

Age (years old), Tertile 1: 20–55, Tertile 2: 56–67, Tertile 3: 68–85; BMI((kg/m^2^)), Tertile 1: 15.20–27.02, Tertile 2: 27.03–32.37, Tertile 3: 32.38–84.40; RBC count (million cells/uL), Tertile 1: 2.78–4.31, Tertile 2: 4.32–4.74 Tertile 3: 4.75–7.55; The subgroup analysis was adjusted for model 3 except effect modifier. DII, Dietary Inflammatory Index; CI, Confidence Interval; BMI, body mass index; RBC, Red blood cells.

## 4 Discussion

More findings (12, 13, 27) demonstrated that the diets of RA patients had a higher inflammatory potential than healthy people. Diet plays an important role in regulating inflammation, but this has not been sufficiently emphasized in current treatment plans for RA. There are few studies on how to determine the effect of diet on inflammatory status in RA patients by immune-inflammatory biomarkers. Our study fills a gap in this area by using DII to assess the clinical significance of SII, NLR, PLR, and LMR among patients with RA. A total of 2,500 RA patients were enrolled in this study. The restricted cubic spline models revealed that SII and NLR are near-linear related to DII. The generalized linear mixed model further displayed that SII and NLR are independently associated with DII. Interestingly, a significant interaction effect was detected (*p* < 0.05), leading to contrasting β values at distinct red blood cell levels. However, the association between DII and PLR was not observed in the generalized weighted multiple linear regression model. Although a significant positive correlation was observed between DII and LMR in the unadjusted model, such correction was not statistically significant when the covariates were taken into account. Therefore, our finding suggests that pro-inflammatory dietary status in RA patients is significantly positively correlated with SII and NLR, potentially influenced by red blood cell levels.

This study identified an association between a pro-inflammatory diet and elevated NLR and SII in RA patients. To our acknowledgments, compared to other inflammatory markers, the sensitivity and specificity of NLR and SII to assess the inflammatory activity are both relatively high (28–30). In a study evaluating the clinical efficacy of TNF-α inhibitors for rheumatoid arthritis, SII and lymphocytes demonstrated the highest predictive value in the DeLong test compared to traditional inflammatory markers (31). A cross-sectional observational study (32) found that the efficacy of NLR is comparable to that of CRP and it is not impacted by the cytokines influencing CRP and erythrocyte sedimentation rate (ESR). Neutrophils are the primary effector cells during the pannus formation in rheumatoid arthritis, while lymphocytes are inflammatory regulators, which accumulate at the sites of inflammatory joints and might result in a decreased lymphocyte count in peripheral blood in patients with RA. Although monocytes and platelets also have regulatory effects on the immune system, neutrophils are more active in the regulation of inflammation than them (9). Therefore, SII and NLR have a better value than PLR and LMR for assessing RA disease activities, which has been proved by another study (30). Furthermore, higher DII and blood inflammation indicators have been shown to synergistically increase the risk of developing cognitive impairment (33) and cataracts (34). We demonstrated a strong association between pro-inflammatory diet and SII/NLR in patients with RA, which was not relatively reliable in PLR and LMR. The correlation between pro-inflammatory diet and SII/NLR has also been confirmed in some inflammation-induced diseases (35, 36), and they can accurately reflect the inflammatory changes in the diet of RA patients. A case–control study (21) enrolled 100 newly diagnosed cases with RA and health-matched controls showed that patients with the highest DII had significantly higher serum inflammation (hs-CRP and TNF). This result should be expected, as DII scores are based on the effects of diets on inflammatory biomarkers (IL-4, TNF-α, IL-1β, IL-6, IL-10, and CRP). Phenolic compounds in pro-inflammatory diets inhibit cycloxygenase-2 (COX-2) protein expression and prostanoid production with the rise of inflammatory markers such as CRP and ESR, which can lead to inflammatory chronic joint diseases (22). In addition, an increase in insulin resistance might be a plausible pathway that a pro-inflammatory diet is associated with inflammatory markers. Higher consumption of high-calorie food like butter has been shown to increase systemic inflammation by increasing levels of high-sensitivity CRP, E-selectin and soluble vascular cell adhesion molecule-1, which are then responsible for increasing insulin resistance (37). The elevated chemotaxis and infiltration of neutrophils involved in the composition of SII and NLR as core immune cells and the increase in the reactive oxygen species caused by a pro-inflammatory diet might be the main reason for the association between elevated SII/NLR and DII in RA patients. Glutamine is consumed at the highest rate by neutrophils compared with other immune cells (38). The metabolic product of omega-3, resolvin, was also found able to attenuate inflammation and relieve joint pain via the inhibition of neutrophil recruitment in RA (39). N-acetylcysteine could remove reactive oxygen species and inhibit the synthesis of pro-inflammation cytokines, thus reducing the recruitment of neutrophils (40). To some extent, this explains why we observed associations only with SII and NLR.

We found that NLR and SII kept a stronger association with DII when RBC levels in RA patients were 2.78–4.31 × 10^12^/L. It’s been reported that the disease activity of RA can be increased significantly with anemia (41, 42). Although the pathogenesis of inflammatory anemia is not completely understood, cytokines play an important role in impairing erythroid progenitor growth and hemoglobin production in developing erythrocytes. Therefore, RA patients with anemia are more sensitive to diet-related inflammatory indicators, which explains the results of this study.

There are a couples of strengths in the study that enhance the reliability and practicability of the findings. First of all, the use of data from the authoritative survey NHANES allows for the generalizability of the results to the broader American population. In addition, the large sample size and survey period of up to almost 20 years contribute to the robustness of the study. Secondly, the weighted analysis across the trisection of DII generates comprehensive characteristics representative of the US population. Then, three weighted generalized linear regression models were carried out, revealing a more credible correlation after adjusting for potential covariates. Finally, subgroup analysis provides valuable insights into a stronger connection between DII and SII/NLR across anemic populations.

The novelty of our study lies in revealing the new biomarkers to evaluate an anti-inflammatory diet for RA treatment, which can be easily realized by blood tests. Based on our findings, in terms of RA patients with anemia whose inflammatory activity is more sensitive to dietary patterns, more attention should be paid to the improvement of their anemia and dietary status.

This study had some limitations. First, because the study had a cross-sectional design, it was not possible to infer causal relationships between dietary and blood inflammatory indicators, and rheumatoid arthritis. Second, we only included RA patients in the study, so the bias caused by disease independence cannot be excluded. More populations involved in inflammatory diseases should be expanded. Third, assessing DII solely based on one-time dietary recall may not be representative of patients’ daily dietary patterns, and further clinical randomized controlled trials are needed to corroborate our results.

## 5 Conclusion

Our study determined dietary inflammatory potential is significantly positively related to SII and NLR among the RA populations, which was less reliable in PLR and LMR. It’s worth noting that such an association between pro-inflammatory dietary status and SII/NLR among RA patients is influenced by variations in red blood cell levels and is more pronounced in anemic patients. We should advocate healthy and anti-inflammatory diet patterns in RA patients, especially those with anemia to achieve better inhibit inflammation.

## Data availability statement

The original contributions presented in the study are included in the article/supplementary material, further inquiries can be directed to the corresponding author.

## Ethics statement

The studies involving humans were approved by The NHANES study procedures were reviewed and approved by the NCHS Ethics Review Board. Informed consent was obtained from all subjects involved in NHANES. The studies were conducted in accordance with the local legislation and institutional requirements. The participants provided their written informed consent to participate in this study.

## Author contributions

ZL: Writing – original draft, Writing – review & editing. ZX: Validation, Writing – original draft. KS: Writing – original draft. XW: Writing – original draft. EF: Writing – original draft, Writing – review & editing.
